# Time resolved DNA occupancy dynamics during the respiratory oscillation uncover a global reset point in the yeast growth program

**DOI:** 10.15698/mic2014.09.166

**Published:** 2014-09-01

**Authors:** Cornelia Amariei, Rainer Machné, Viktor Stolc, Tomoyoshi Soga, Masaru Tomita, Douglas B. Murray

**Affiliations:** 1 Institute for Advanced Biosciences, Keio University, Tsuruoka, Yamagata 997-0017, Japan.; 2 Systems Biology Program, Graduate School of Media and Governance, Keio University, Fujisawa, Kanagawa 252-8520, Japan.; 3 Institute for Theoretical Biology, Humboldt University, Berlin, Invalidenstrasse 43, D-10115, Berlin, Germany.; 4 Institute for Theoretical Chemistry, University of Vienna, Währingerstrasse 17, A-1090, Vienna, Austria.; 5 NASA Ames Research Center, Moffett Field, California, United States of America.

**Keywords:** respiratory oscillation, chromatin dynamics, transcription regulation, histone modification, anabolism, catabolism, energetics

## Abstract

The structural dynamics of chromatin have been implicated in the regulation of fundamental eukaryotic processes, such as DNA transcription, replication and repair. Although previous studies have revealed that the chromatin landscape, nucleosome remodeling and histone modification events are intimately tied into cellular energetics and redox state, few studies undertake defined time-resolved measurements of these state variables. Here, we use metabolically synchronous, continuously-grown yeast cultures to measure DNA occupancy and track global patterns with respect to the metabolic state of the culture. Combined with transcriptome analyses and ChIP-qPCR experiments, these paint an intriguing picture where genome-wide nucleosome focusing occurs during the recovery of energy charge, followed by clearance of the promoter regions and global transcriptional slow-down, thus indicating a nucleosome-mediated “reset point” for the cycle. The reset begins at the end of the catabolic and stress-response transcriptional programs and ends prior to the start of the anabolic and cell-growth transcriptional program, and the histones on genes from both the catabolic and anabolic superclusters are deacetylated.

## INTRODUCTION

Chromatin is an array of nucleosomes comprising of DNA (~147bp) wound around histone octamers, which pack up to 90% of the DNA 1. This packaging has a structural role by allowing compaction of DNA in the nucleus, which represses transcription as it hinders the binding of transcription factors and transcriptional machinery to gene promoters and coding regions. Thus, the dynamic regulation of eukaryotic chromatin structure has been shown to be central to gene transcription, DNA replication and repair. As chromatin biology is highly conserved in eukaryotes, much of the work in elucidating it has been done using *Saccharomyces cerevisiae.*

While nucleosome positioning is partially determined by the DNA sequence 2,3,4, energy-dependent processes such as nucleosome remodeling 5,6,7 and RNA Polymerase II elongation 8,9,10 play key roles in shaping and maintaining the nucleosomal landscape. The first nucleosome downstream of the transcriptional start site (TSS) and the upstream nucleosome-free region stand out as a target of regulation for nucleosome remodeling complexes, where H2A.Z inclusion, covalent histone modifications and transcription factors facilitate the entry of RNA Polymerase II (PolII) into the gene 7,11,12,13.

Previous studies have produced high-resolution maps of nucleosome positioning 4,14,15 and investigated genome-wide chromatin remodeling and histone modifications using chemical and/or genetic perturbations 7,16,17,18. Mutants of nucleosome remodelers or general transcription factors can have drastic effects on differential promoter architectures 16. Even subtle changes in histone binding affinity and nucleosomal positioning have large repercussions on the phenotype, e.g., shifting a nucleosome by a few base pairs at a promoter can lead to marked changes in the transcription rate 5,7. Genes that differ in nucleosome configurations and are differentially affected by nucleosome remodelers encode for global cellular processes such as cell growth (ribosomes, anabolism), mitochondrial growth and catabolism or the cellular stress response 14,19.

The essentiality of these processes and the highly coupled feedbacks involved in their regulation make it very difficult to experimentally establish causality of global regulatory interactions* in vivo, *and any alteration of the system invariably results in pleiotropic phenotypes. For example, the effects of modifying cellular ATP concentrations by mutation or chemical inhibition will percolate throughout cellular physiology, changing every aspect of the study. Global effects stemming from changes in energetic levels and direct causal relations between these changes and specific cellular processes are almost impossible to delineate *in vivo*. Instead, one needs to find correlations *in vivo* and try to understand them in terms of mechanistic relations known, with particular reference to less complex *in vitro* biochemistry. The impact of cellular energetics on chromatin structure is probably best exemplified by the strict requirement of ATP, but not of transcription or replication, for *in vitro* reconstitution of *in vivo*-like nucleosome configurations at promoters 6.

**Figure 1:**
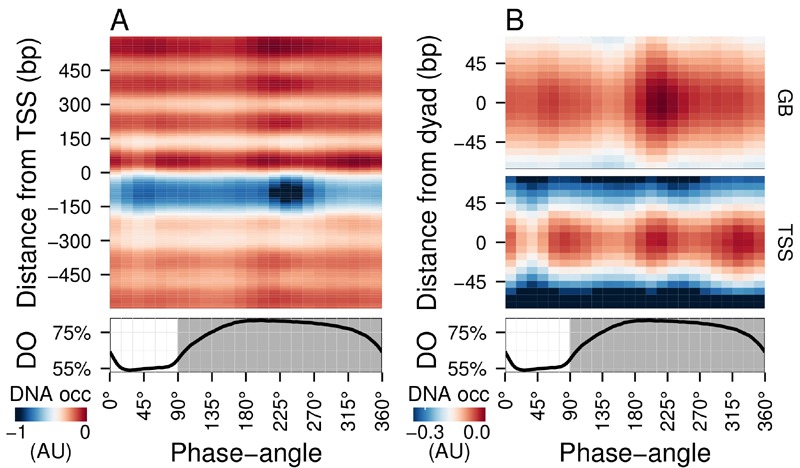
FIGURE 1: Average DNA occupancy dynamics around TSS during one respiratory cycle. An average cycle was constructed by a cubic spline fitting of the dataset (normalized by the least variant set 49) comprising three respiratory cycles (33 samples; 6 min sampling; oscillation period 67 min), where protein-bound and genomic DNA sample pairs were extracted using our one-pot method. The median DNA occupancy (DNA occ = log2(pDNA) - log2(gDNA) - MNase bias; see Methods; capped at -1 in A for increased resolution) was calculated for 5140 genes aligned to the transcriptional start site (**(A)**; TSS; 0 bp) and aligned to putative nucleosome dyads (**(B)**; 0 bp) defined by 4. Putative nucleosomes were further classified according to their position with respect to TSS as gene body nucleosomes (GB; except for +1/terminal nucleosomes) and TSS nucleosomes. Time is represented as phase-angles calculated according to the respiratory oscillation (represented as a subscript throughout the text), where the minimum first derivative of the residual dissolved oxygen data (bottom panels) represents 0°/360°. Dark grey marks the reductive phase. Median nucleosome profiles at upstream and terminal nucleosomes are shown in Figure S2.

When *S. cerevisiae* is grown under precisely controlled continuous culture conditions, individuals auto-synchronize physiological events to produce a respiratory oscillation 20,21,22. This experimental system is highly stable and reproducible, allowing physiological dynamics to be probed at a very high temporal resolution. Additionally, diverse datasets obtained at different times and from different laboratories can be reliably correlated. In this mode of culture growth, cells alternate between an oxidative phase and a reductive phase (Fig. 1), which is conveniently monitored by the residual dissolved oxygen concentration (DO). DO is low during the oxidative phase (high oxygen uptake rate) and high during the reductive phase (low oxygen uptake rate). Importantly, ATP:ADP shows up to a 7 fold change each cycle, peaking during the oxidative phase 19,23, and the majority of mRNA species have an oscillatory, phase-dependent transcription 24,25,26. A comparative analysis of two distinct oscillatory transcriptome datasets 19 revealed two super-clusters of co-expressed genes, one primarily expressed in oxidative phase, whose gene products are involved in cell growth and anabolism (anabolic supercluster), and the other expressed in the reductive phase, whose products were primarily involved in mitochondrial growth and function, stress response and catabolism (catabolic supercluster). Each of the superclusters comprised a temporal transition between gene cohorts 14, with differential nucleosome configurations at promoters and gene body in terms of positioning, occupancy and size of the NDR (nucleosome-depleted regions), and differential effects on nucleosome configurations of global transcription factor mutants and ATP-dependent nucleosome remodelers 5,16. Here, we correlate DNA occupancy dynamics, mRNA expression timing and the metabolic state of the culture. Protein occupancy at NDR-flanking nucleosomes increases at the transitions between high and low energy states, coinciding with the transitions between the expression superclusters. A rapid sequence of genome-wide events occur after energy state has reached a minimum during the reductive phase, i.e., a genome-wide increase of histone occupancy (a "nucleosome focusing" event), clearance of protein occupancy at the NDR, and a global decrease in transcription rates. After this sequence, ATP:ADP recovers quickly and the expression of anabolic supercluster genes proceeds. These time-resolved data imply the observed nucleosome focusing event as a key step that resets transcription during the respiratory oscillation.

## RESULTS

### Genome-wide multiphasic DNA occupancy dynamics

Time-series samples of protein-bound micrococcal nuclease (MNase) digested DNA (pDNA; for digestion profiles see Fig. S1) and genomic DNA (gDNA) were hybridized to tiling arrays. Fluorescent signals from each channel were used to calculate the logarithmic base 2 ratios for each probe. These ratios then had the MNase bias (calculated from triplicate microarray hybridizations; see Methods) subtracted to calculate the DNA occupancy time-series (Fig. 1). Spatially, the average DNA occupancy profiles around the transcriptional start sites (TSS; Fig. 1A) follow the canonical pattern of a NDR centered at ~75 bp upstream of TSS, flanked by well organized nucleosome arrays. Temporally, a Fourier analysis of the dataset revealed the major period (67 min; 15% of probes; p-value < 0.05), but it is immediately apparent that the DNA occupancy data comprises additional dynamics over one metabolic cycle (Fig. 1A). However, the resolution of the tiling arrays used made identification of individual nucleosomes unreliable. Therefore, we used a genome-wide high-resolution map of *S. cerevisiae* nucleosomal dyads 4 and cross-referenced these with our dataset, to define preferential nucleosomal positions.

Nucleosomes found +2 to the penultimate TSS-covering nucleosome were defined as gene body nucleosomes (Fig. 1B, GB) and showed two peaks per cycle (GB_60°_ and GB_225°_), while all nucleosomes found in NDR-flanking regions at both 5' and 3' ends of genes (Fig. S2), including the TSS-covering nucleosome, show three peaks (Fig. 1B, TSS_75°,_ TSS_225°_ and TSS_330°_). TSS_225°_ and the major GB_225°_ events coincide, whereas TSS_75°_ and TSS_330°_ coincide with the metabolic transitions between oxidative and reductive phases. The NDR is maintained throughout the cycle, but the DNA occupancy at the NDR is lowest during two temporal windows (45° and 240°; Fig. 1A). Thus, DNA occupancy events occurring during the respiratory oscillation are dynamic and multiphasic, where occupancy events TSS_330°_ and TSS_225° _and GB_225°_ at the nucleosomal regions are followed by a marked decrease in protein occupancy at the NDR.

### DNA occupancy dynamics are only weakly correlated with differential transcript expression

**Figure 2:**
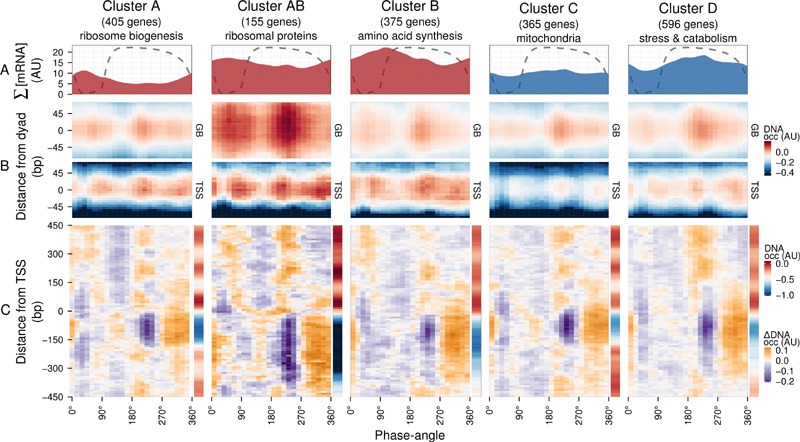
FIGURE 2: Expression dynamics of major gene clusters and the DNA occupancy dynamics at their average gene promoters during a respiratory cycle. Messenger RNA abundances from the consensus clusters (Clusters A, AB, B, C and D; 19) from a time-series microarray dataset 26 (48 samples; 4 min sampling, period 50 min) were summed for each cluster **(A)**, ∑[mRNA]. Transcripts that belong to the anabolic supercluster (A, AB and B; produced during the oxidative phase) and those that belong to the catabolic supercluster (C and D; produced during the reductive phase) have a solid red and blue fill, respectively. The median DNA occupancy profiles of the aligned nucleosome dyads for the consensus clusters during a respiratory cycle (**(B)**, DNA occ) were calculated as in Figure 1B. The DNA occupancy dynamics (**(C)**, ΔDNA occ) were normalized by subtracting the log-ratio temporal average (red and blue side bar) from the median DNA occupancy for each expression cluster (see Fig. 1A). Dotted lines represent the residual dissolved oxygen (DO), scaled to the y-axis range of the panel. The transcript and nucleosome datasets were aligned using the minimum and maximum first derivative of the DO (Fig. S4A). The minimum first derivative of the DO data represents 0°/360°. The differential expression clusters B.C and B.D were merged into an expanded cluster B, as these were co-expressed during the short period oscillation. These clusters and lower signal-to-noise ratio clusters are shown in Figure S3.

To explore DNA occupancy data in the context of gene expression, we compared the dataset with the temporal program of co-expressed and functionally coherent gene cohorts during the respiratory oscillation (Fig. 2) 19. Clusters A, AB and B are representative of the anabolic supercluster, whose sum of transcripts (∑[mRNA]; Fig. 2A) peaked during the oxidative phase, while clusters C and D represent the catabolic supercluster that peaked during the reductive phase. Surprisingly, we could observe little difference in the temporal profiles of DNA occupancy between the genome average at nucleosomal positions and the gene clusters (Fig. 2B and Fig. S3C), except for a relatively higher occupancy for anabolic supercluster genes at GB_60°_, when they are being transcribed, and a broader GB_225° _peak for catabolic genes, during their expression maxima, which supports data that remodeling correlates at best weakly with the timing of transcriptional activation 27. The differences in overall intensity between clusters can be attributed to the average nucleosome occupancy profiles (sidebars in Fig. 2C and Fig. S3D), which are consistent with previous analyses, i.e., genes comprising of clusters A and C have well-positioned nucleosomal arrays and relatively short NDR, genes comprising clusters B and D have fuzzier nucleosomal arrays both upstream and downstream, and AB (ribosomal proteins) genes are characterized by very large upstream NDR and prominent but fuzzy nucleosomal arrays 19.

The normalized occupancy profiles (Fig. 2C and Fig. S3D main panels) reveal a biphasic DNA occupancy in the NDR, completely antiphase with gene body nucleosomes. The maximum promoter occupancy (330°) coincides with the highest occupancy at TSS (TSS_330°_), at the onset of respiratory activity (transition to oxidative phase), when transcript levels of the anabolic clusters increase. The other phase transition point, where the transcript abundances of catabolic clusters rises, is also marked by an increased TSS occupancy (TSS_75°_). Interestingly, the global peak at 225° coincides with a transient increase in transcript abundances of all gene clusters (Fig. 2A and Fig. S3A and B; global maxima for catabolic and local maxima for anabolic clusters). This genome-wide event is quickly followed by a striking depletion in DNA occupancy at the promoter NDR (240°), visible in all clusters, and a transcript abundance minimum (global minima for anabolic and local minima for catabolic clusters; 270°; Fig. 2A and Fig. S3A and B).

Therefore, a global chromatin restructuring event during the respiratory oscillation occurs similarly for all genes regardless of promoter architecture. While the three TSS events (Fig. 2A and Fig. S3C) correspond to increases in ∑[mRNA] globally (Fig. 2B and Fig. S3A), they are not reflected in the differential expression profiles of the anabolic and catabolic superclusters.

### Tracking histone occupancy, histone acetylation and metabolic state during the respiratory oscillation

**Figure 3:**
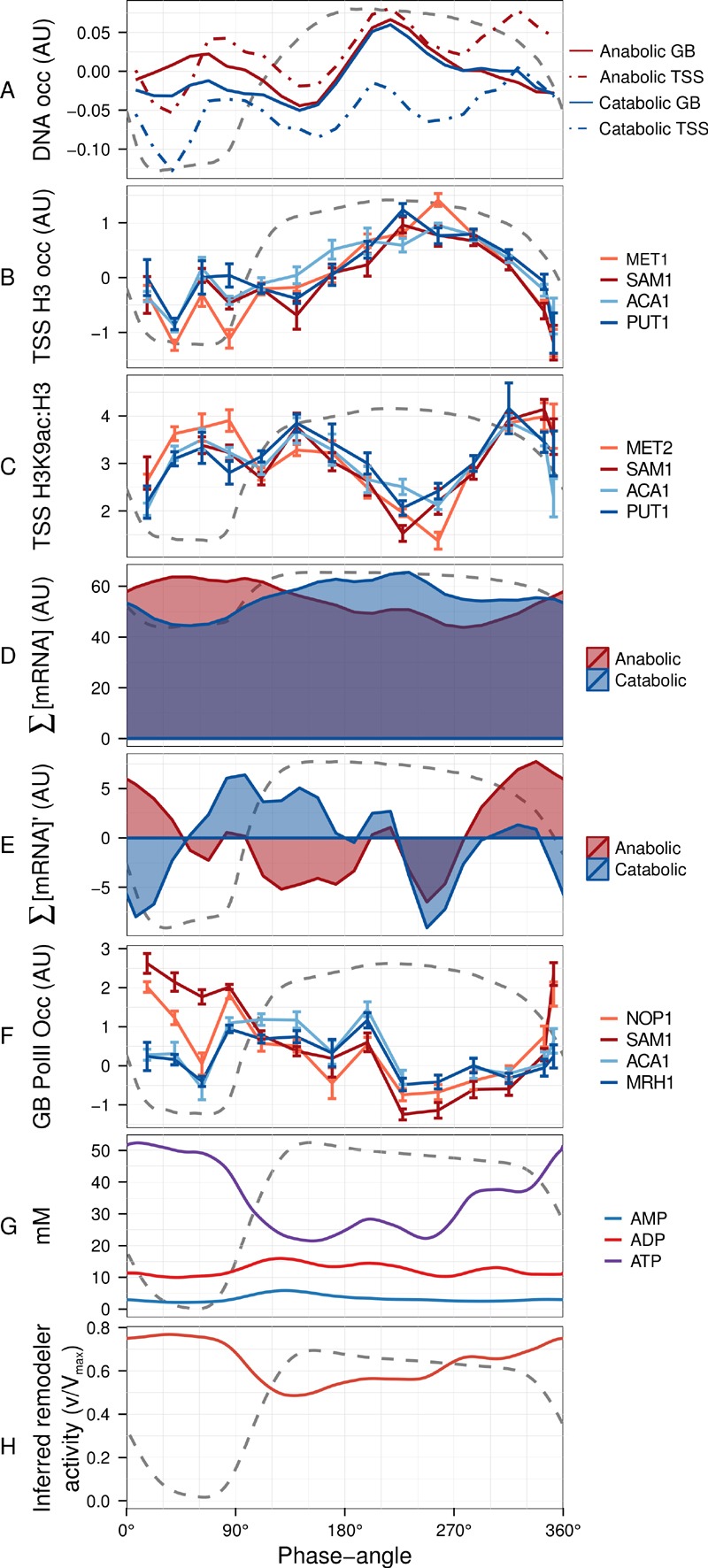
FIGURE 3: Defining the reset point of the respiratory oscillation. The temporal profile of the DNA occupancy (DNA occ) at the center of the nucleosome region for the anabolic and catabolic superclusters **(A)** were calculated as in Figure 1B. Histone H3 **(B) **and H3K9ac (**(C)**, log-ratio values with respect to H3) ChIP time-series (14 samples; 4 min sampling; period 53 min) were amplified by qPCR at TSS regions of 4 representative anabolic (red hues) and catabolic (blue hues) genes. Total mRNA abundances for each supercluster (**(D)**, ∑[mRNA]) were used to calculate mRNA abundance rate of change (**(E)**; ∑[mRNA]'; change in mRNA abundance every 15°). The same samples for B and C were used for RNA PolII ChIP-qPCR at genome body regions of 4 representative anabolic (red hues) and catabolic (blue hues) genes **(F)**. The PolII signals were normalized with respect to a subtelomeric region on chromosome VI. ATP, ADP and AMP concentration time-series data (**(G)**, 36 samples, 6 min sampling, period 78 min, Fig. S4B) measured by capillary electrophoresis mass spectrometry 48 were used to calculate inferred ISWI remodeling rates **(H)**. An average cycle was constructed by a cubic spline fitting for each time-series spanning several cycles (A, D, E, G, H). Error bars in B, C, F represent standard error of mean of qPCR triplicates. Dotted lines represent the residual dissolved oxygen (DO), scaled to the y-axis range of each panel, and datasets were aligned using the minimum and maximum first derivative of DO concentration (Fig. S4A). The minimum first derivative of the DO data represents 0°/360°. The anabolic supercluster was defined as clusters A, AB, B, B.C, B.D and ab.n and the catabolic supercluster was defined as clusters C, D, cd.n and cd.ab.

To further characterize these genome-wide events in promoter and TSS occupancy, we tested histone H3 presence and, following the reported histone acetylation involvement in the initiation of anabolic program 28, the acetylation state (H3K9ac:H3) by ChIP-qPCR at the TSS of two anabolic and two catabolic genes (Supplementary Methods, primers in Table S1). Again, we find at best subtle differences between differentially transcribed genes but clear correlations to the global occupancy dynamics (Fig. 3A-C). TSS H3 occupancy gradually increases over the reductive phase in all genes and its peak coincides with the global event (GB_225°_ and TSS_225°_), not exhibiting major secondary peaks. In contrast, H3K9 acetylation is enriched at the two transition events that are seen at TSS and promoter-flanking sites (TSS_75°_ and TSS_330°_), suggesting these peaks in TSS protein occupancy may rather reflect binding of promoter-remodeling or transcription initiation complexes rather than an increase histone occupancy. Notably, a large decrease in acetylation during the mid reductive phase (210°-270°) coincides with the nucleosomal focusing event, providing evidence that this event was a result of histone deacetylation.

The total transcript levels of the two superclusters (∑[mRNA]; Fig. 3D) were used to calculate global changes in transcript turnover (first derivative ∑[mRNA]'; Fig. 3E). This revealed a short increase, then sharp drop of ∑[mRNA]' in both superclusters at the nucleosome focusing point, potentially reflecting a co-release of transcripts with PolII and a subsequent global halt on transcription, or increased RNA degradation. These transcription rate profiles closely matched the PolII occupancy in the gene body at two anabolic and two catabolic genes (Fig. 3F, Supplemental Methods, primers in Table S2), confirming that a decrease in transcriptional activity contributes to the slowdown.

To interpret these events in terms of cellular energetics, we monitored ATP during the respiratory oscillation (Fig. 3G and Fig. S4B). Bulk cellular ATP concentration changes (21-51 mM) correlate with anabolic supercluster abundance changes (∑[mRNA]'; Pearson correlation *r *= 0.54, p-value = 0.005; Fig. 3E, 3G and Fig. S4B) and strongly anti-correlate with the catabolic supercluster transcription (*r* = -0.86, p-value = 8.23x10^-8^, Fig. 3E, 3G and Fig. S4B), indicating that ATP concentration may play a pivotal role in the differential transcription of the catabolic and anabolic superclusters. The changes in ATP, EC (0.65-0.9) and ATP:ADP (1.2-8) ratios were large and will have marked influence on many enzymes that require ATP as a cofactor. We therefore used the measured ATP and ADP concentrations, to infer the remodeling activity for ISWI (Fig. 3H), calculated assuming a competitive inhibition and the reported *in vitro* ATP Michaelis constant of 0.15 mM 29 and an ADP inhibition constant of 0.1 mM 30, is at a minimum when catabolic genes are expressed maximally. The inhibition of ISWI is thought to involve the binding of ADP in between the DNA and the complex, causing the affinity to decrease 30. Interestingly, ATP also causes a similar effect on mutants with no ATP hydrolysis activity. The exact mechanism is still to be fully elucidated, and competitive inhibition will perhaps underestimate the influence of low energy states on remodeling activity, as mixed inhibition is likely. However, our inferred activity (0.5-0.75, where 1 is 100% activity) illustrates that changes in ATP availability in enzymes with product inhibition, such the ISWI complex, will show large changes in activity. Furthermore, it supports a defining role for ATP availability in nucleosome remodeling events 19.

In summary, global DNA occupancy dynamics correlate with a global slowdown in transcription and suggest a reset point for the respiratory cycle, where nucleosomes are focused, the NDR becomes well-defined and proteins disassociate.

## DISCUSSION

Mechanistically, finding cause-and-effect relationships is difficult in complex oscillatory systems with multiple feedback loops and experimental determination of causality in global regulation of growth is very difficult *in vivo*. Altering ATP or the affinity of chromatin remodeling by mutation irrevocably alters the cellular network structure, because of the centrality of the processes under examination 31, e.g., a *gts1*Δ mutant that is involved in energy metabolism and the regulation of ADP-ribosylation 32 caused an unstable chaotic oscillator with multiple periods 33 and pleiotropic phenotypes. Therefore, we approach these issues by accumulating time resolved datasets from a highly reproducible experimental system, then derive correlations, and interpret these in the light of previously published data and mechanistic relations, primarily determined from *in vitro* biochemistry.

Using this approach, we show that the repression of general transcription during the reductive phase of the respiratory oscillation is preceded by rapid a sequence of global events. A decrease in ATP concentration and energy charge goes hand-in-hand with an up-regulation of the catabolic supercluster genes. Next, a global nucleosome focusing event (GB_225°_ and TSS_225°; _Fig. 1 and Fig. 3) occurs, and data suggests this event involves increased H3 occupancy and deacetylation (Fig. 3). Shortly after this the DNA occupancy of the promoter regions drastically decrease and PolII dissociates from the coding regions, which results in the observed global transcriptional slowdown. We suggest that the nucleosome focusing event represents a transcriptional "reset" point, where nucleosomal arrays become well-positioned because of the greater affinity of deacetylated histones for DNA. Such a chromatin state has been achieved *in vitro *6 by adding cell extract and ATP (no other nucleotide triphosphates were required), indicating that ATP-dependent remodeling activities alone are sufficient for defining a well-positioned nucleosomal landscape. In our system, ATP has a minor peak (210°-240°) during the reset event, suggesting that ATP-dependent remodeling is involved during this *in vivo* chromatin reorganization.

A similar global dissociation at the promoters has been previously observed in the hypoosmotic stress response in yeast 34. Additionally, the common stress response in yeast has a high degree of overlap with the catabolic supercluster, thus implicating this reset point in its development. Indeed, for a range of stress responses up to a 4 fold increase in survivability was observed around the reset point 35. The implications of these observations are profound, especially in the context of heterogeneity and persistence of pathogens and cancer cells. Considering that cells in populations are usually desynchronized and that the metabolic oscillation has been shown to occur in individuals 36, the resistance to drugs in subpopulations may stem from this transcriptionally-inactive stress-resilient state, hardwired into the chromatin dynamics during the cellular growth program.

Additionally, TSS regions show two peaks of protein-occupancy (TSS_75°_ and TSS_330°_), which coincide with shifts in ATP concentration and mark the increase in transcription of the catabolic and anabolic superclusters, respectively. While the lowest concentration of bulk ATP (25 mM; Fig. 3G) is much higher than the *in vitro* ATP Michaelis constant (K_m_) for the chromatin remodeling complexes RSC (K_m_ = 0.08 mM) 37, INO80 (K_m_ = 0.143 mM) 38 and ISWI (K_m_ = 0.15 mM) 29, the heterogeneity of the cell environment (e.g., gradients, compartments and molecular crowding 39) may lead to nuclear concentrations of ATP that are significantly lower than the bulk measurements, i.e., within the nuclear compartment ATP concentration will tend to approach the K_m_. Moreover, K_m_ determination in *in vitro* simplified reaction conditions yields values that can be an order of magnitude different than found in the *in vivo *complex reaction matrix, where effectors are known to antagonize remodeler activity 40. *In vitro*, ADP is known to inhibit chromatin remodelers 30,41 and the inferred remodeling rates (Fig. 3H) for ISWI support the idea that cellular energetics can have a strong influence on chromatin state *in vivo,* and that the differential ∑[mRNA]' for catabolic and anabolic superclusters (Fig. 3E) may stem from two short phases of promoter-restructuring by nucleosome remodeling and histone modifying enzymes at the TSS nucleosomes. Previously, we found differential effects of repressive (Isw2 5) and activating (RSC 16) ATP-dependent remodelers 19 on nucleosome configurations, which may provide a mechanistic basis for how these similar occupancy events develop into the differential expression of the anabolic and catabolic superclusters, where alternating ATP availability would flip transcription from the anabolic to the catabolic program. The activities of these enzymes may regulate transcription of the supercluster genes differentially, e.g, by incorporation of H2A.Z 12,13, histone acetylation 28 and lateral sliding of TSS nucleosomes 5,7, and the activities of these enzymes will be the focus of future investigations.

Taken together, our data points to global mechanisms of gene regulation, starting with a general reset of chromatin architecture and continuing sequentially through a defined temporal program, where cellular growth and DNA replication are, by necessity, flexibly coupled 24,36,42,43. In this context, the chromatin reset point can be interpreted as a metabolic equivalent of cell-cycle start, where the chromatin structure itself is a major sensor of the metabolic state.

## MATERIALS AND METHODS

Unless otherwise stated all chemicals and reagents were supplied by Wako Pure Chemical Industries, Ltd (Japan).

### Culture growth

The *S. cerevisiae* strain used was IFO 0233 and was grown in continuous culture as previously described 44.

### Protein-bound and genomic DNA purification; a one pot method 

Samples (1 mL) were fixed in formaldehyde (1% v/v) and placed on a rotator (5 rpm, room temperature, 12 min), then neutralized with 0.1 volume of 2 M glycine and placed again on a rotator (5 rpm, room temperature, 12 min). Samples were then centrifuged (5200 g, room temperature, 1 min) and the supernatant was discarded. The pellets were stored at -80°C.

Cross-linked samples were thawed on ice, washed twice in 1 mL TBS and pelleted at 12000 g, 4°C, 2 min. Pellets were resuspended in 500 μL of Zymolyase digestion solution (50 mM Tris-HCl pH 7.4, 0.07% 2-Mercaptoethanol, 1000 U/mL of Zymolyase 100T) and incubated on a rotator (5 rpm, room temperature, 1 h). MNase (microccocal nuclease) digestion mix containing Igepal (0.1%), spermidine (200 μM), NaCl (50 mM), MgCl_2 _(5 mM) and CaCl_2 _(1 mM) were added to the tubes to the final concentrations that are shown in parentheses. MNase (150 U) and RNase A (5 μL; Promega, Japan, catalogue number: A7973) were then added, and samples were incubated (37°C; 30 min). The NaCl, MgCl_2_, CaCl_2_ and MNase were omitted if genomic DNA was required. To stop the digestion, tubes were transferred to an ice bath then EDTA and SDS were added to a final concentration of 0.01 M and 0.05%, respectively.

Each sample was then centrifuged (12000 g, 4°C, 10 min), the supernatant was transferred, and 10 μL Proteinase K was added (20 μg in 100 μL of nuclease free water; Roche, Japan). After an overnight incubation (65°C, ~10h) the reaction mixture was transferred to 550 μL phenol:chloroform:isoamyl alcohol 25:24:1 in phase-lock tubes (MaXtract Low Density, Quiagen, Japan), and centrifuged (12000 g, room temperature, 5 min). The aqueous phase was transferred to a new tube in which ethanol (2 v/v), glycogen (0.04 v/v) and 5 M NaCl (0.04 v/v) were added. After centrifugation (12000 g, room temperature, 30 min), the supernatant was discarded and pellet was washed twice with 1 mL 80% ethanol. The pellet was then resuspended in 100 μL water and purified using QIAquick PCR purification kit (Qiagen) according to the manufacturer's instructions. The final samples were eluted in 100 μL nuclease-free water and stored at 4°C. DNA was quantified and fragment sizes were inspected (Fig. S1).

MNase control samples were prepared by mixing 20 μL of purified genomic DNA sample with 480 μL with 50 mM Tris-HCl pH 7.4, and MNase digestion mix was added as above. After the addition of MNase (3 U), samples were incubated (37°C; 10 min), digestion was stopped and samples were purified as before.

### Labeling and hybridization

For each set separately (protein-bound, genomic and MNase control DNA), an equal amount of each sample was used for labeling (the average of the highest quartile of the volume corresponding to 1 μg of DNA in each set), ensuring that most samples will have over 1μg of DNA. Genomic samples were labeled with Cy3 and protein-bound DNA samples were labeled with Cy5, according to the labeling kit instructions (NimbleGen Dual-Color Labeling Kit, Roche, Japan, catalog number: 06370270001). MNase control samples were labeled with Cy3. The resulting samples were hybridized according to the NimbleGen Arrays user's guide (ChIP-chip Arrays, version 6.2) on custom-made, NASA Ames Research Center yeast 360K whole genome tiling arrays 45, washed (NimbleGen Wash Buffer Kit, Roche, Japan, catalogue number: 05584507001) and scanned on an Agilent Microarray Scanner (G2565BA, Agilent, Japan) according to the manufacturer's instructions.

### Measurement of NAD(P)H fluorescence and adenosine nucleotide concentrations

As NADH and NADPH concentrations are difficult to extract and quantify due to binding with macromolecules and rapid oxidation 46, we used a relative, live cell measure of combined NAD(P)H fluorescence 47. AMP, ADP and ATP were extracted by bead-beating and measured by CE-MS, cell number and volume were measured by electronic cell counter (CDA500, Sysmex, Japan) and metabolite concentrations calculated to mM 48.

The relative remodeling activity (v/V_max_) at a Michaelis constant *K_m_* and an ADP inhibition constant *K_i_*was calculated using the competitive product inhibition formula:

**Figure:**
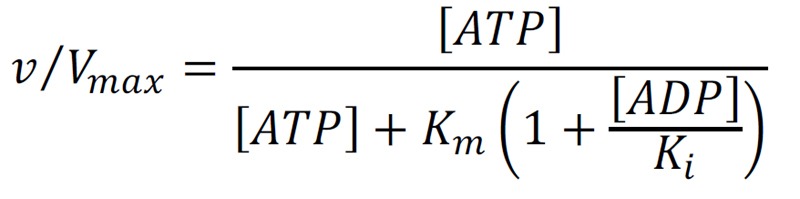


### Data acquisition and normalization

NimbleScan software (Roche) was used to extract raw data from the dual color tiling array image files in the “.pair” file format, for individual channels, according to the user guide (version 2.6). The dataset contained 33 time-series pairs of protein-bound DNA (pDNA) and genomic DNA (gDNA) signals, and 3 MNase-digested controls (mDNA). The normalization between arrays was based on the Least Variant Set 49,50. 24916 probes (6.35%) with a variance across arrays lower than 0.1 in the pDNA and gDNA datasets were used to construct a LOESS fitting curve for individual arrays and subsequently used for normalization on each channel. The threshold of 0.1 was chosen because it produced the most coherent signal while preserving the temporal structure of the data. The arrays contained paired forward and reverse strand probes, which were averaged at this step. Finally, the MNase bias was calculated as:

**Figure:**
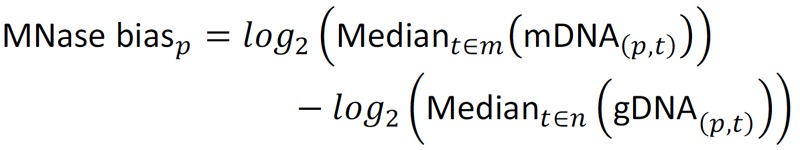


where *m *represents the mDNA arrays, *n* represent gDNA arrays, and was incorporated in the final calculations of DNA occupancy at each probe *p* on array *t, *defined as:

**Figure:**
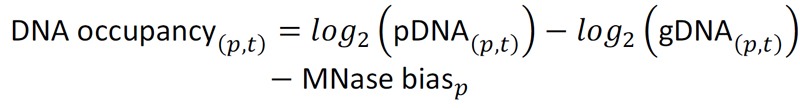


It should be noted that these values are all relative, therefore are not quantitative. The mean DNA occupancy was -0.18 with a standard deviation of 0.67.

### Genome build and calculations

The features considered were taken from the SGD genome annotation file (http://downloads.yeastgenome.org/curation/chromosomal_feature/saccharomyces_cerevisiae.gff 2005-11-05; genome build: sacCer3, 2011_02_03, R64-1-1). A nucleotide BLAST against this build was performed to define the positions of all probes on the tiling array.

The transcription start site positions for 5176 genes were taken from Machné and Murray, 2012 and references therein and adjusted to this genome build.

### Phase angle and oscillation statistics relative to the DO time-series

DO data (sampled at 0.1 Hz) were smoothed by a running mean with a window of 0.01 Hz, and the result was used to calculate the first derivative. The minimum and maximum first derivative of the DO data were used to define the start of the oxidative and reductive phases, respectively. Due to difference in the length of the oxidative and reductive phases, we aligned all data by setting as all minima to 0° and all maxima to 94° and phase adjusting each sample point to these references linearly (Fig. S4A).

Phase-angle and amplitude and signal-to-noise ratio statistics were calculated as previously described 19,44,51.

### Data smoothing and interpolation

To obtain the average temporal profile during one respiratory cycle (between 0° and 360°), the experimental values with respect to their phase-angles were smoothed with a cubic spline (degrees of freedom corresponded to the number of samples per respiratory cycle in the dataset). The resulting spline was resampled at 24 points (every 15°). The change rates for each dataset were calculated as the difference between each two consecutive data-points (prior to phase-angle calculations), and assigned the average of the two sample phase-angles, after which the same smoothing and resampling methods were used. The NAD(P)H time-series, which had a sampling frequency of 10 Hz, was converted from time-domain to frequency-domain DFT, and frequencies lower than the frequency of the respiratory oscillation and those higher than 4 times the frequency of the respiratory oscillation were removed. The filtered signal was reconstructed by the inverse transform.

### Footnotes

The data discussed in this publication have been deposited in NCBI's Gene Expression Omnibus 52 and are accessible through GEO Series accession number GSE60112 (http://www.ncbi.nlm.nih.gov/geo/query/acc.cgi?acc=GSE60112 ).

## SUPPLEMENTAL MATERIAL

Click here for supplemental data file.

All supplemental data for this article are also available online at http://microbialcell.com/researcharticles/time-resolved-dna-occupancy-dynamics-during-the-respiratory-oscillation-uncover-a-global-reset-point-in-the-yeast-growth-program/.
