# Supplementary material for: Time resolved DNA occupancy dynamics during the respiratory oscillation uncover a global reset point in the yeast growth program
Source: Microb Cell. 2014 Sep 1;1(9):279–88. doi: 10.15698/mic2014.09.166 (PMC5349131; doi:10.15698/mic2014.09.166)
Supplement: Supplementary file 1 [file mic-01-279-s01.pdf]

SI

Materials and methods

Chromatin Immunoprecipitation and qPCR

The frozen pellets were thawed on ice, washed in FA-lysis buffer (50 mM HEPES-KOH pH 7.5, 140 mM NaCl, 1mM EDTA, 1% Triton X-100, 0.1% Na Deoxycholate, 1 mM PMSF, 1 μg/mL leupeptin, 1 μg/mL pepstatin), resuspended in 700 μl of the same buffer and disrupted by bead-beating, using 0.5 mm diameter glass beads and beat for 5x 30 seconds with 1 minute cooling breaks on ice. After removing the beads, 150 μl of the cell lysate was diluted in FA-lysis buffer to a volume of 1.5 mL and sonicated for 15 min (cycles of 30 s on and 30 s off). Finally, the sonicated samples were centrifuged twice and the supernatant was stored at -80°C.

For each ChIP, 75μL of chromatin extract was transferred into 225μL FA-lysis buffer to a Protein LoBind microcentrifuge tube (Eppendorf, Japan) and incubated with 2μL of antibody for 3h to overnight at 4°C on a rotary wheel. The IP reaction was centrifuged (12000 g, 4°C, 10 min) and the supernatant was transferred to a Corning Costar Spin-X tubes (Sigma-Aldrich, Japan) containing 30μl of protein A agarose beads slurry (Pierce, Japan; pre-incubated with 1 mg/mL BSA). The samples were incubated on a rotary wheel (0.025 g, 4°C, 2 h) and centrifuged (900 g, 4°C, 2 min). The elute was discarded and the column was washed sequentially (5 min each) with 700μL of FA-lysis buffer, FA500 (50 mM HEPES-KOH pH 7.5, 500 mM NaCl, 1 mM EDTA, 1% Triton X-100, 0.1% Na Deoxycholate), LiCl wash solution (10mM Tris-HCl pH 8.0, 250 mM LiCl, 1 mM EDTA, 0.5% NP-40, 0.5% Na Deoxycholate) and TES (10mM Tris-HCl pH 7.4, 1 mM EDTA, 100 mM NaCl). The column was transferred to a tube and incubated with agitation (150 rpm, 37°C, 30 min) after adding 100μL of elution buffer (100mM Tris-HCl pH 8, 10 mM EDTA, 1% SDS, 400 mM NaCl). The sample was eluted by centrifugation (7500 g, room temperature, 2 min) and treated with Proteinase K (20 μg in 100 μl of nuclease free water; Roche, Japan) 65°C overnight. The sample was purified using the QIAquick PCR purification kit (Qiagen, Japan) following the manufacturer instructions. The resulting DNA samples were stored at -20°C.

For total chromatin control samples (inputs), 0.75 µL of chromatin extract was mixed with 100 µL of elution buffer, treated with Proteinase K and purified as described above. The antibodies used were: anti-PolII (ab5408; Abcam, Japan) and anti-H3 (ab1791; Abcam, Japan).

For RT-qPCR reactions, 2 µL of each sample was amplified by real-time PCR using Absolute QPCR SYBR green reagents (Abgene, Japan), with the primer sets in Tables S1 and S2 for RNA PolII and H3 experiments, respectively. Due to issues in creating suitable primers for the promoter regions of *MRH1* and *NOP1*, they were swapped with *MET2* and *PUT1*, which have similar transcription profiles.

DNA Quantification

DNA concentrations were quantified by the Hoechst 33258 method (http://www.biotek.jp/resources/articles/dna-hoechst-33258.html). 0.5 µL sample was dissolved in 99.5 µL of Hoechst solution (0.1 µg/mL Hoechst 33258; Dojindo, Japan), 10 mM Tris base, 1 mM Na_2EDTA, 200 mM NaCl, into 99.5 µL water (UltraPure™, DNase/RNase-Free Distilled Water, Life Technologies, Japan) loaded on a black 96-well plate (Greiner Bio-one, Japan, catalog number: 655076) and read using a multi plate reader (excitation 360 nm; emission 460 nm; SpectraMax, Gemini XS, Molecular Devices, USA). The DNA concentration was calculated relative to a ladder of 5-162.5 ng HIND III digest of phage lambda DNA (Toyobo, Japan).

For RT-qPCR experiments, 5 µL of 1:10 dilutions of both genomic DNA and protein-bound DNA samples were amplified using SYBR Premix Ex Taq II (Takara, Japan) on a DNA Engine Opticon system (Bio-Rad, Japan).

Other computational methods

The arrays contained probes with a length of 36bp, with a median resolution of 42bp across the yeast genome. The DNA occupancy profile along each chromosome was constructed by aligning probes to corresponding chromosomal coordinates and filling the spaces between adjacent probes with linear values[1]. If the distance between two probes was higher than 50bp, the values were discarded. Nucleosome boundaries based on published dyad positions[2] were decided as either

73bp away from a dyad, or at the mean distance between two dyads, when the inter-dyad distance was less than 147bp. The index (Idx) of each nucleosome dyad (*d*) was calculated with respect to the distance in bp to the nearest TSS as follows: $\mathrm{Idx}_{d,d>(\mathrm{TSS}-25)} = \lfloor \frac{(d-\mathrm{TSS}+24)}{162} + 1 \rfloor$; $\mathrm{Idx}_{d,d<(\mathrm{TSS}-147)} = \lfloor \frac{(d-\mathrm{TSS}+147)}{162} + 1 \rfloor$; $\mathrm{Idx}_{d,(d-\mathrm{TSS})\in[-147,-25]} = 0$. These cutoffs for 0 and +1 were chosen at the local minima in the distribution of the dyad positions with respect to TSS. Terminal nucleosomes (TN) were classified by counting backwards from the last Idx identified in each gene. The genome annotation file contained 6603 ORFs (including 810 dubious, 897 uncharacterized and 4896 verified genes), 21 pseudogenes, 299 tRNA, 27 rRNA, 77 snoRNA, 6 snRNA and 15 ncRNA.

The adenylate energy charge (EC) was calculated by the formula[3]:

$$EC = \frac{[ATP] + 0.5[ADP]}{[AMP] + [ADP] + [ATP]}$$

Bibliography

1. Flores O and Orozco M (**2011**). nucleR: a package for non-parametric nucleosome positioning. **Bioinformatics** 27(15): 2149–2150. doi: 10.1093/bioinformatics/btr345.

2. Brogaard K, Xi L, Wang J-P, and Widom J (**2012**). A map of nucleosome positions in yeast at base-pair resolution. **Nature** 486(7404): 496–501. doi: 10.1038/nature11142.

3. Ball WJ and Atkinson DE (**1975**). Adenylate energy charge in Saccharomyces cerevisiae during starvation. **J Bacteriol** 121(3): 975–982. .

4. Machné R and Murray DB (**2012**). The yin and yang of yeast transcription: elements of a global feedback system between metabolism and chromatin. **PLoS One** 7(6): e37906. doi: 10.1371/journal.pone.0037906.

5. Li CM and Klevecz RR (**2006**). A rapid genome-scale response of the transcriptional oscillator to perturbation reveals a period-doubling path to phenotypic change. **Proc Natl Acad Sci U S A** 103(44): 16254–16259. doi: 10.1073/pnas.0604860103.

Supplemental Figures

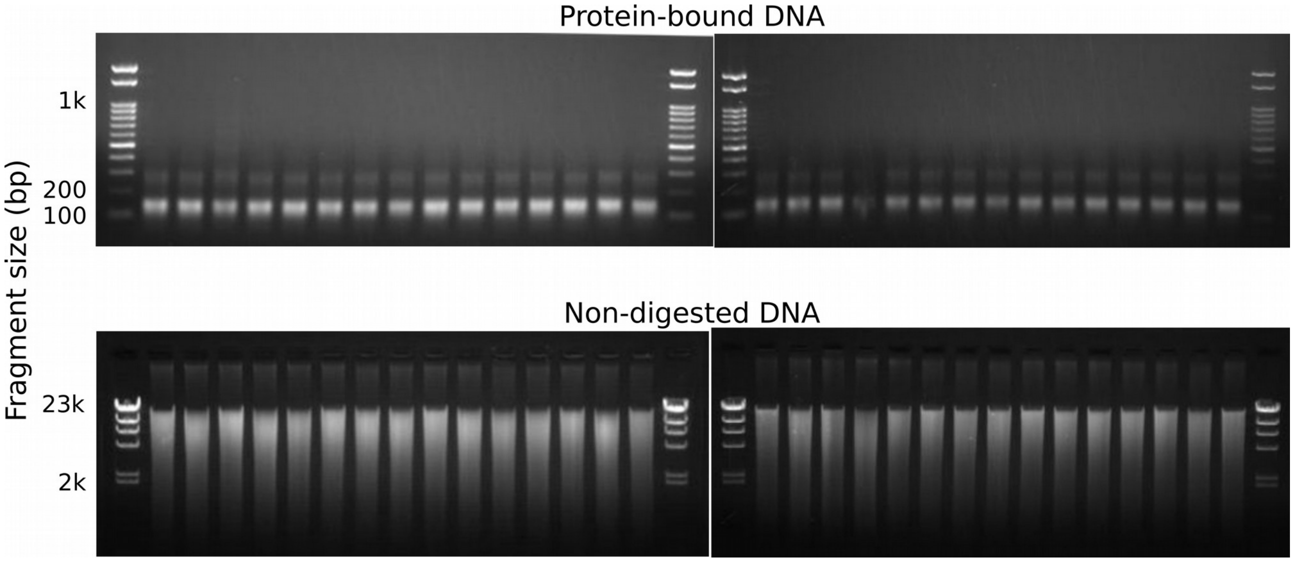

Figure S1. Protein-bound and genomic DNA extraction. MNase-digested samples (top) yielded on average 8.2 µg DNA/mL of sample culture and were resolved on a 1.5% agarose gel. Genomic DNA samples (bottom) yielded on average 19.7 µg DNA/mL of sample culture and were resolved on a 0.9% agarose gel.

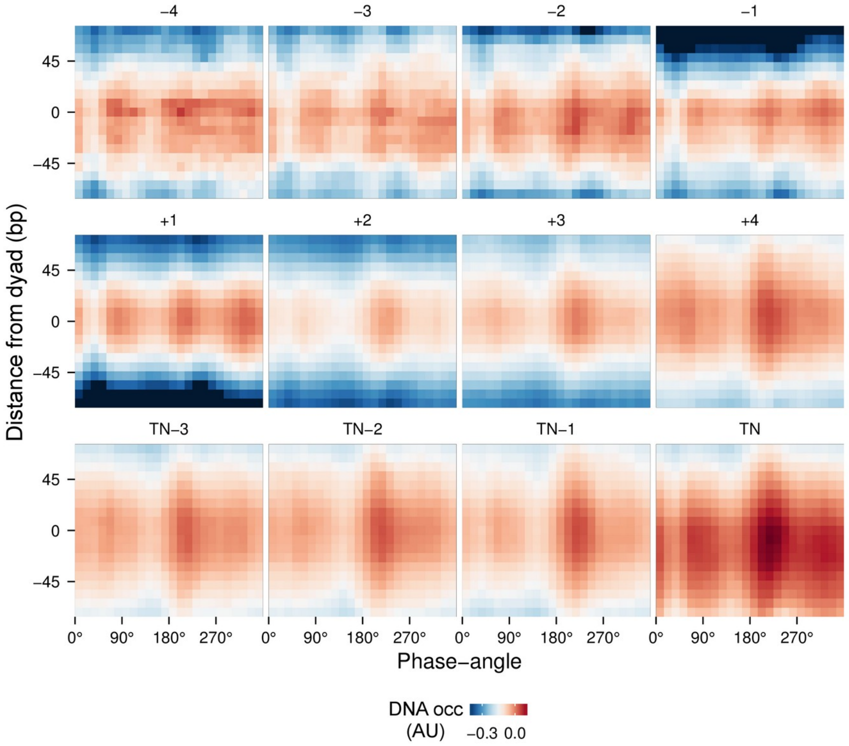

Figure S2. DNA occupancy along an average gene at different nucleosomal positions. The median DNA occupancy (DNA occ = log2(pDNA) – log2(gDNA) – MNase bias; see methods) was calculated for data aligned to nucleosome dyads (data from [2]), which were classified according to their position with respect to TSS (-4 to +4) and stop codons (TN-3 to TN, supplemental methods), covering the average length of a gene (~8 nucleosomes). Upstream nucleosomes (-4 to -1) show the same temporal profiles as the TSS and terminal nucleosomes (+1, TN). The +2, TN-1 nucleosomes shows a transitional temporal profile from the flanking nucleosomes to the rest of the body core nucleosomes (+2 to +4, TN-3 to TN-2), still showing a trough at 45° but without a peak at 330°. Nucleosomal 0 positions were omitted due to low signals. Upstream nucleosomes that intersect other genomic features were removed from the analysis. See Figure 1 for a description of the dataset.

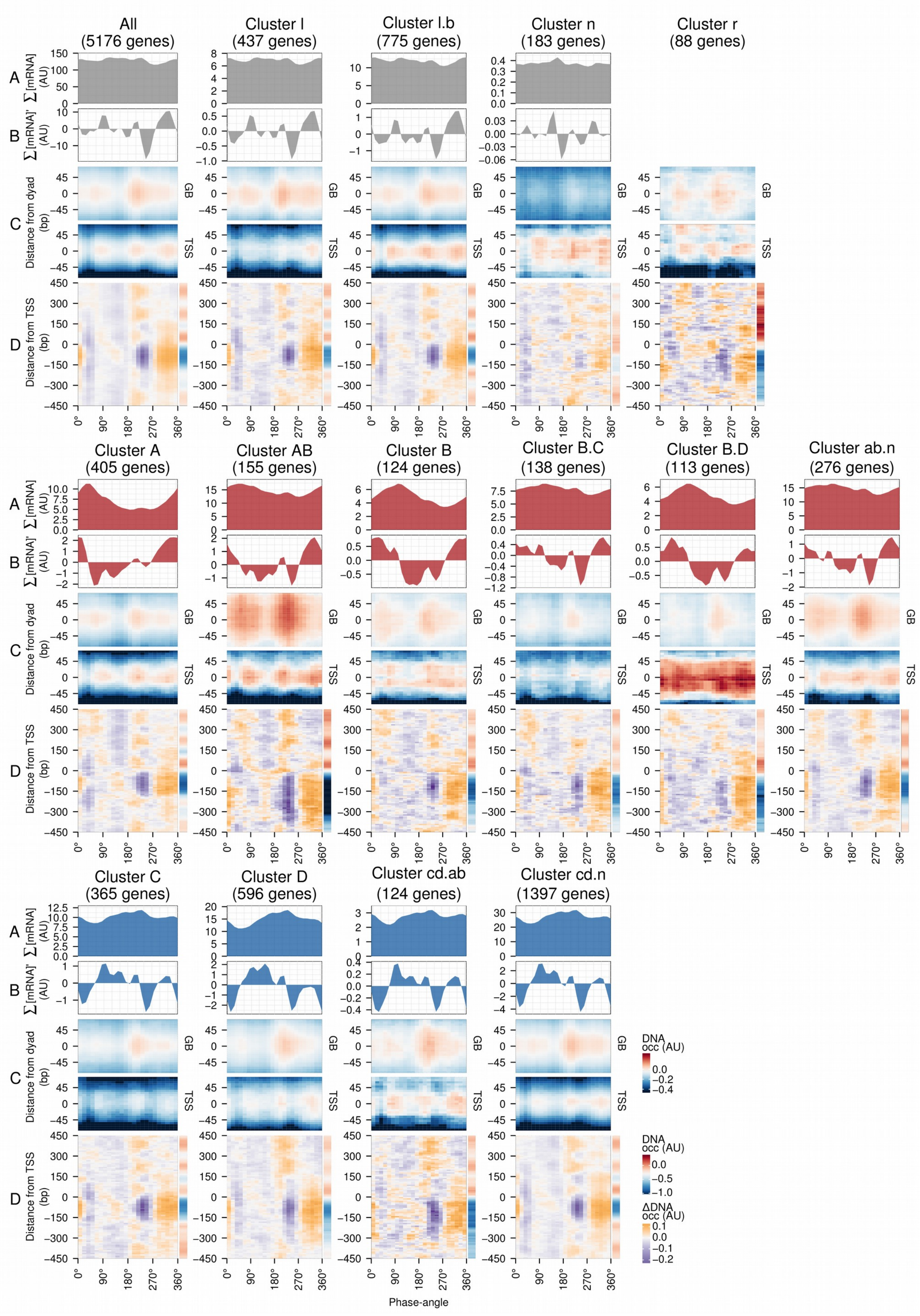

Figure S3. Expression dynamics of all gene clusters and the DNA occupancy dynamics at their

average gene promoters during a respiratory cycle. Messenger RNA abundances from all expression clusters[4] (data from [5]) were summed for each cluster (A; ∑[mRNA]), and panels were split in rows according to their expression timing: low signal-to-noise (grey), anabolic (red) and catabolic (blue). mRNA expression data for cluster r genes was not available. mRNA abundance rate of change (B; ∑[mRNA]') were calculated as the change in mRNA abundance (A) every 15°. The median DNA occupancy (DNA occ) aligned to nucleosome dyads comprising each cluster (C) was calculated as in Fig. 1B. The DNA occupancy dynamics (ΔDNA occ; D) were normalized by subtracting the log-ratio average of each position (side bar) from the data presented in Figure 1A. Dotted lines represent the DO, scaled to the y-axis range of the panel. The two datasets were aligned using the minimum and maximum first derivative of the DO (Fig. S4A). The minimum first derivative of the DO data represents 0°/360°. All the transcripts (All) and low amplitude ("noisy") clusters (l, l.b, n and r), transcripts that belong to the anabolic supercluster (A, AB, B, B.C, B.D and ab.n; produced during the oxidative phase) and those that belong to the catabolic supercluster (C, D, cd.ab and cd.n; produced during the reductive phase) have a solid grey, red and blue fill, respectively. For a full description of the clustering approach see [4].

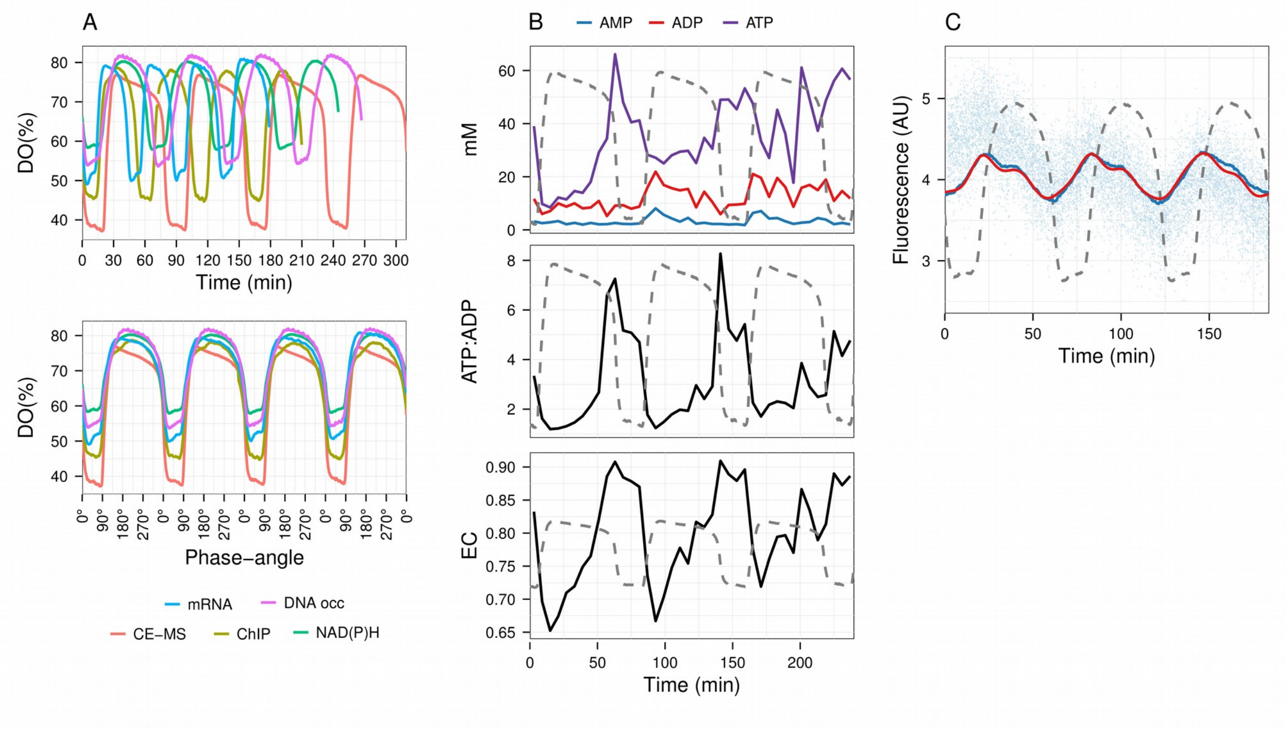

Figure S4. Dataset alignment and raw time-series data. Residual dissolved oxygen (DO) time-series from the five experimental datasets taken from separate continuous culture runs (A, top). Time-series samples were taken for adenosine nucleotide concentrations using capillary electrophoresis mass spectrometry (CE-MS; this study), combined fluorescent emission signal for NADH and NADPH (NAD(P)H; this study), mRNA expression tiling arrays (mRNA; data from [5]), DNA occupancy tiling arrays (DNA occ; this study) and chromatin immunoprecipitation quantitative polymerase chain reaction (ChIP; this study). Phase-angle calculations and alignment (A, bottom) were performed by using the minimum and maximum first derivative of the dissolved oxygen trace. CE-MS measured concentrations (B, top) of AMP (blue), ADP (red) and ATP (purple) were used to calculate energy charge (EC, B, middle) and ATP:ADP (B, bottom). NAD(P)H measurements (C, light blue dots, measured every 10ms), were smoothed with a running mean (RM, dark blue) and with a Fourier filter signal (red) for comparison. RM is the difference between the running mean with a window of 1.2 minutes and a second running mean with a window of 60 minutes. Corresponding dissolved oxygen traces for figures B and C are shown in grey dashed lines were scaled to the range of the panel.

Table S1. Primer sets used for ChIP-H3

Gene	5' Primer	3' Primer
ACA1	CGACTAAAAGCGCAGAAACTGTATT	TCGTTACCTGGTGTTATTCCAGTACT
MET2	CTTGTTCACGGATATTTCTTGCTTT	GAGAAACTTTAGACGGACCCTGTG
PUT1	AAATCGCATGAACTAAGCCCA	GAGAAGAGCTGCTACGGACTTGT
SAM1	TTGGGACGTATATATCGACTGGTG	TTTTTAGGGTAAATTCCTGGTTTTTACT

Table S2. Primer sets used for ChIP-PolII

Gene	5' Primer	3' Primer
ACA1	GCTGCAATGACCCCGTATG	TCGCCAGATGGGATAGAAGG
MRH1	ACGTTTTGCAGCCAGACTCTG	CCGAAGTGGTTAGCAATTGGA
NOP1	TCAGATCTAAGTTGGCTGCCG	AGAAGTACCGGAAGCAGCACC
SAM1	CTGAATCCGTTGGTGAAGGTC	CGCAACTTTGGAGTGAGGGT
subTEL VI*	TAACAAGCGGCTGGACTACTTTC	GATAACTCTGAACTGTGCATCCACTC
